# A Node Generation and Refinement Algorithm in Meshless RPIM for Electromagnetic Analysis of Sensors

**DOI:** 10.3390/s25041115

**Published:** 2025-02-12

**Authors:** Zihao Li, Siguang An, Guoping Zou, Jianqiang Han

**Affiliations:** College of Mechanical and Electrical Engineering, China Jiliang University, Hangzhou 310018, China

**Keywords:** adaptive algorithms, domain decomposition, radial basis function, meshless method

## Abstract

In sensor design, electromagnetic field numerical simulation techniques are widely used to investigate the working principles of sensors. These analyses help designers understand how sensors detect and respond to external signals during operation. One popular method for electromagnetic field computation is the meshless radial point interpolation method (RPIM), where the number and distribution of nodes are critical to ensuring both accuracy and efficiency. However, traditional RPIM methods often face challenges in achieving stable and precise results, particularly in complex electromagnetic environments. In order to enhance the stability and accuracy of electromagnetic numerical calculations, a node generation and adaptive refinement algorithm for the meshless RPIM is proposed. The proposed approach includes an initial node-generation method designed to optimize the balance between computational accuracy and efficiency, as well as a dynamic error threshold and hybrid node refinement method to precisely identify and adaptively refine areas requiring additional nodes, ensuring high precision in critical regions. The proposed method was validated through its application to electrostatic fields and multi-media magnetic fields, demonstrating significant improvements in both stability and accuracy compared with conventional RPIM approaches. These findings highlight the potential of the proposed algorithm to enhance the reliability and precision of electromagnetic field simulations in sensor design and related applications.

## 1. Introduction

In recent years, electromagnetic simulation has become increasingly important in sensor design. Modern sensors often contain complex magnetic and electronic components, making the understanding of magnetic field distribution within these components critical [1]. This understanding helps optimize the sensor’s sensitivity, power consumption, and overall performance. Sensors are typically composed of multiple components, and simulating how the electromagnetic field from one component affects others is essential. Magnetic elements, such as inductive coils, Hall effect sensors, and magnetoresistive materials, are at the core of many sensor operating principles [2,3]. Accurate electromagnetic simulation is crucial for predicting how these components interact with each other and with external magnetic fields. Without such simulations, it would be extremely difficult to design these components for optimal performance, as physical testing alone cannot deeply explore the complex electromagnetic behavior within the sensor.

Electromagnetic compatibility (EMC) is another key factor that requires support from electromagnetic simulation [4]. As electronic devices become smaller and more powerful, they often operate in environments with higher levels of electromagnetic noise. Therefore, sensors must be designed to function reliably even in the presence of electromagnetic interference. Electromagnetic simulation enables the optimization and customization of sensor designs to meet specific requirements. It provides flexibility in design adjustments, allowing simulations to refine designs to achieve desired performance goals.

For this reason, high-quality electromagnetic computational algorithms are vital to the sensor design process. These algorithms play a critical role in ensuring the sensor’s optimal functionality and performance in real-world conditions. Meshless methods have been widely used in solving electromagnetic problems [5,6,7,8]. Among these methods, the meshless radial point interpolation method (RPIM) has emerged as a popular branch due to its simplicity and flexibility. The RPIM facilitates interactions between interpolation nodes distributed within the computational domain, whether uniformly or randomly, instead of relying on a mesh structure [9]. As a result, the need to consider mesh quality is eliminated in the RPIM, providing advantages in solving electromagnetic problems. Given that in the RPIM, calculations are based on the interpolation nodes, the number and location of the nodes are critical. A significant challenge for the RPIM lies in precise node generation, both in terms of quantity and distribution. An excessive number of nodes will lead to an ill-conditioned and irreversible coefficient matrix with a high condition number [10], while an insufficient number of nodes may decrease the computational accuracy. Therefore, an effective and efficient node-generation method is crucial for the RPIM.

Endeavors have been made to alleviate the condition number of the coefficient matrix and enhance the computational accuracy of the RPIM. The domain decomposition method is utilized to divide the computational domain into sub-domains, resulting in a sparse global coefficient matrix that facilitates the identification of the proper shape parameter. Additionally, domain decomposition can mitigate the impact of large condition number by reducing the number of degrees of freedom in the computational domain. When the entire domain is divided into multiple sub-domains, each sub-domain problem is transformed into a boundary value problem by imposing boundary conditions at the artificially created interfaces [11,12]. A lower condition number reduces the system’s sensitivity to perturbations in the input data [13]. Furthermore, computational accuracy can be improved by adding additional nodes [14].

However, existing methods often struggle to achieve a balance between computational efficiency and accuracy, particularly in complex electromagnetic environments with multi-media interfaces [15]. Domain decomposition and node-addition techniques may introduce new challenges, such as sudden jumps (Riccati-type) in electromagnetic fields, which can significantly impact the accuracy of numerical simulations. These discontinuities, which arise from the dynamic nature of electromagnetic fields, can lead to abrupt changes in field components, making it difficult to achieve precise results in affected domains [16,17]. To address these limitations, this study proposes an initial node-generation method and an adaptive node refinement algorithm. The proposed approach introduces a dynamic error threshold and a hybrid node refinement strategy, ensuring optimal node distribution and significantly improving the stability and accuracy of the RPIM in complex electromagnetic simulations.

## 2. Implementation of RPIM for Electromagnetic Computation

### 2.1. Radial Point Interpolation Method

A typical electromagnetic field problem is generally represented by a governing equation in the form of partial differential equations [10]:

(1) $$\left\{\begin{array}{c} L(u(x))=f\;in\Omega \\ B(u(x))=g\;on\Gamma \end{array}\right.$$

where, Ω represents the computational domain, while Γ denotes the boundary, *L* is the linear partial differential operator, *B* is the boundary condition, and *f* and *g* are known functions of the independent variable *x*.

By placing nodes throughout the entire computational domain and establishing the function space at each node, the unknown coefficients *u*(*x*) in the computational domain can be obtained:

(2) $$u(x)\approx u^{h}(x)=\sum_{j=1}^{N}a_{j}\phi_{j}(x),$$

where *u^h^*(*x*) represents the approximate unknown coefficients, and *a_j_* are the coefficients to be determined. This paper chooses the multiquadric (MQ) radial basis function for its superior performance; the MQ-RBF is expressed as follows:

(3) $$\phi_{j}(x)={\left({\left\|x-x_{j}\right\|}^{2}+c^{2}\right)}^{0.5},$$

From the above equation, a system of *N* linear equations can be obtained for the unknown coefficients *a_j_*:

(4) $$\left\{\begin{array}{cccc} \phi_{1}(x_{1}) & \phi_{2}(x_{1}) & \cdots & \phi_{N}(x_{1})\\ \phi_{1}(x_{2}) & \phi_{2}(x_{2}) & \cdots & \phi_{N}(x_{2})\\ \vdots & \vdots & \ddots & \vdots \\ \phi_{1}(x_{N}) & \phi_{2}(x_{N}) & \cdots & \phi_{N}(x_{N}) \end{array}\right\}\left[\begin{array}{c} a_{1}\\ a_{2}\\ \vdots \\ a_{N} \end{array}\right]=\left[\begin{array}{c} u_{1}\\ u_{2}\\ \vdots \\ u_{N} \end{array}\right],$$

The above equation can be expressed in matrix and vector notation, where

(5) $$K=\left[\begin{array}{c} \Phi^{T}(x_{1})\\ \Phi^{T}(x_{2})\\ \vdots \\ \Phi^{T}(x_{N}) \end{array}\right]=\left\{\begin{array}{cccc} \phi_{1}(x_{1}) & \phi_{2}(x_{1}) & \cdots & \phi_{N}(x_{1})\\ \phi_{1}(x_{2}) & \phi_{2}(x_{2}) & \cdots & \phi_{N}(x_{2})\\ \vdots & \vdots & \ddots & \vdots \\ \phi_{1}(x_{N}) & \phi_{2}(x_{N}) & \cdots & \phi_{N}(x_{N}) \end{array}\right\},$$

(6) $$\Phi \left(x\right)={\left[\phi_{1}(x),\phi_{2}(x),\ldots ,\phi_{N}(x)\right]}^{T},$$

(7) $$u={\left[u_{1},u_{2},\ldots ,u_{N}\right]}^{T},$$

(8) $$a={\left[a_{1},a_{2},\ldots ,a_{N}\right]}^{T},$$

Therefore, the above equation can be represented as a matrix equation:

(9) Aa=u,

From the above equation, the unknown coefficient vector can be obtained. Substituting it into the equation gives the approximate function expression at any node *x*:

(10) $$u^{h}(x)=\Phi^{T}(x)A^{-1}u=N(x)u,$$

### 2.2. Initial Node-Generation Method

There are two traditional methods for generating initial nodes: random distribution and uniform distribution. However, these two methods may result in an excessive or insufficient number of nodes, which may affect the effectiveness or accuracy of the algorithm. To control the quantity of initial nodes, a structured background grid is introduced here, and the computational domain is covered with equal-sized square grids. Based on the background grid, the number of nodes can be controlled by step length. The step length between two nodes is calculated as follows:

(11) $$l_{step}=\frac{w}{n+1},$$

where *w* is the width of the background grid, *n* is the number of nodes between two vertices on the same side of the grid, *n* is an integer starting from zero and increasing in each iteration until *l_step_* ≤ *l*_0_, and *l*_0_ is the preferred maximum step length defined by the designer.

In the proposed initial node-generation method, the number of nodes can be controlled using step length. The larger *l_step_* is, the fewer nodes are generated. Furthermore, the structured background grid is a decomposition of the computational domain, so the coefficient matrix has the merit of sparsity.

### 2.3. Adaptive Refinement Algorithm

In order to add nodes precisely and improve the accuracy of the RPIM, an adaptive refinement algorithm is proposed. Two-step iterations are used in the proposed node refinement algorithm. The first step is to determine the low-accuracy area; the second step is to add appropriate nodes in this area.

In the first step, a dynamic error threshold is proposed to mark the low-accuracy nodes. In each iteration, the errors of nodes are calculated and classified within the background grid. Maximum error in each background grid is chosen to represent its grid quality. The worst quality background grid with the largest maximum error is marked. Node refinement is performed only within the marked background grid, which helps reduce the complexity of the refinement process. The second largest maximum error is chosen as the error threshold for the current iteration, which ensures that the refinement is effectively confined to the relevant background grid, preventing excessive refinement caused by local anomalous errors. This approach maintains a balance between accuracy enhancement and computational efficiency. In the marked background grid, if the error of the node is larger than the error threshold, the node is marked. The error is calculated according to [18]:

(12) $$ê(x)=\left|\Phi_{h}(x)-\Phi_{c}(x)\right|,$$

where Φ*_h_* and Φ*_c_* are high-precision and current solutions, respectively. In this paper, Φ*_h_* is a solution obtained with 1/2 *l*_0_.

**Figure 1:**
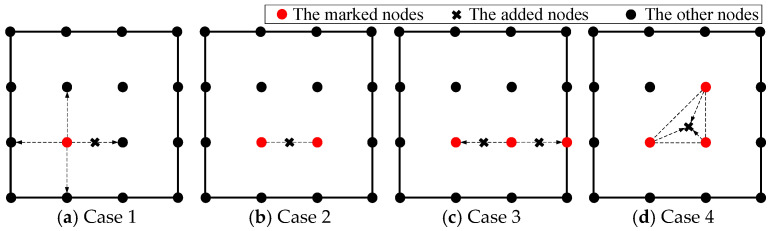
Different node-adding patterns in hybrid refinement method.

In the second step, a hybrid method based on centroid [19] and midpoints [20] refinement is proposed to add nodes around the marked nodes found by the first step. New nodes are generated based on the position and number of the marked nodes. There are four basic node adding patterns, which are shown in Figure 1. The arrow illustrates the process of generating additional nodes through the marked nodes The pseudocode for the hybrid refinement algorithm is presented in Algorithm 1. Lines 1–13 detail the process of marking the precision nodes, while lines 14–38 outline four basic node adding patterns.

Case 1: Only one node is marked. A nearest neighbor search is used to find the closest node to the marked node. If the marked node has equal distances to its neighbor nodes, the node with the largest error is chosen as the closest node. The midpoint between the marked node and its closest node is added, as in Figure 1a and Algorithm 1, lines 15–21.

Case 2: Two nodes are marked. A new node is added directly at the midpoint of the two marked nodes, as in Figure 1b and Algorithm 1, lines 22–24.

Case 3: Three nodes are marked. It is checked as to whether the marked nodes can form a triangle or not. If a triangle cannot be formed, the midpoints of the marked node are generated as adding nodes, as in Figure 1c; otherwise, the centroid point of the triangle is added, as in Figure 1d and Algorithm 1, lines 25–32.

Case 4: More than three nodes are marked. Three of the marked nodes are chosen and then Case 3 is implemented. The node with largest error among the marked nodes is identified and two neighbor marked nodes are located using nearest neighbor search, as in Algorithm 1, lines 15–21.

The newly added node *N_A_* of the midpoint and *N_B_* of the centroid algorithm are defined as follows:

(13) $$N_{A}=(\frac{x_{1}+x_{2}}{2},\frac{y_{1}+y_{2}}{2}),$$

(14) $$N_{B}=(\frac{x_{1}+x_{2}+x_{3}}{3},\frac{y_{1}+y_{2}+y_{3}}{3})$$

**Algorithm 1:** Adaptive Refinement for RPIM1:**Initialize** node and grid distribution, maximum iteration count ***i_max_***2:**While** (iteration < i_max_) **do**3: Calculate errors for each node4: **for** each background grid:5:  ***E_g_*** ← maximum error of each grid6: **end**
**for**
7: Mark the background grid with the largest ***E_g_***8: **Error threshold** ← second-largest ***E_g_***9: **for** each node in the marked background grid:10:  **If** error (node) > error threshold:11:   Mark this node12:  **end**
**if**
13: **end**
**for**
14: ***x*** ← number of the marked node15: **If** x = 1:16:  The closest node ← nearest neighbor search17:  **If** number of the closest node ≠ 1:18:   The closest node ← the closest node with the largest error19:  **end**
**if**
20:  Refined nodes = initial nodes + midpoint (the marked node, the closest node)21: **end**
**if**
22: **If** x = 2:23:  Refined nodes = initial nodes + midpoint (the marked node)24: **end**
**if**
25: **If** x = 3:26:  **If** the three nodes form a triangle:27:   Refined nodes = initial nodes + centroid (the marked node)28:  **end**
**if**
29:  **else**
30:   Refined nodes = initial nodes + midpoint (the marked node)31:  **end else**
32: **end**
**if**
33:**If** x > 3:34:  The marked node with the largest error ← nearest neighbor search35:  Back to 2536: **end**
**if**
37:**end while**38:**Return** refined node distribution

### 2.4. Algorithm Description

**Figure 2:**
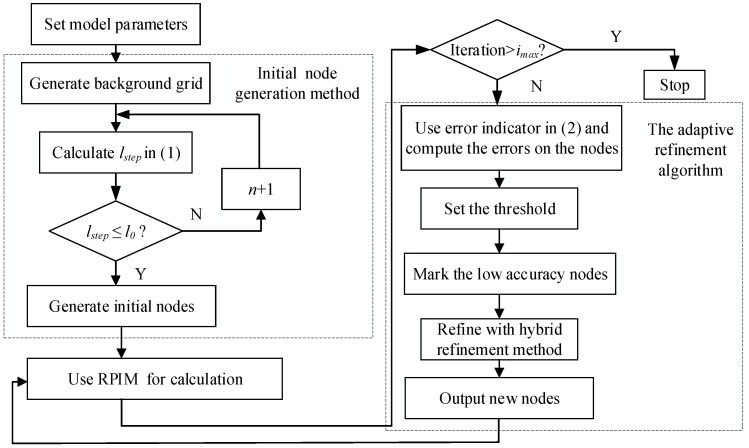
Flow chart of the proposed algorithm.

To demonstrate the proposed algorithm intuitively, a flow chart of the proposed algorithm is shown in Figure 2. The algorithm is explained in detail:

Step 1: Set model parameters, including the size of the domain, object material, and location.

Step 2: Set the minimum gap width between two materials as the width of the background grid.

Step 3: Calculate the *l_step_* using Equation (11) to set uniformly-distributed nodes, and then proceed with the calculation using initial grids and nodes.

Step 4: Compute the errors on the nodes with Equation (12), determine the marked background grid and the error threshold of the current iteration, and determine the marked nodes.

Step 5: Check the number of marked nodes, and generate adding nodes according to the proposed hybrid refinement algorithm.

Step 6: Calculate the results with the new nodes using the RPIM. If the iteration number is higher than the preset iteration number *i_max_*, go to Step 7; otherwise, go to Step 4.

Step 7: Stop the algorithm.

## 3. Numerical Results

To verify the performance of the proposed algorithm, it was implemented using MATLABR2021b to solve electromagnetic problems in electrostatic fields [21] and multi-media magnetic fields [22].

### 3.1. Square Metal Box

**Figure 3:**
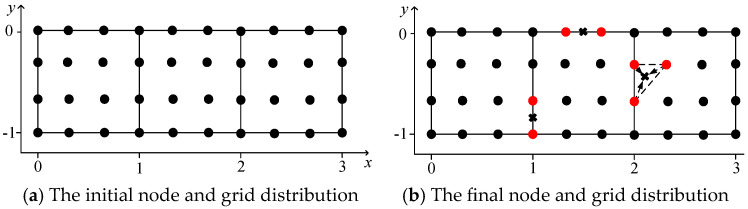
The initial and final node and grid distribution.

**Figure 4:**
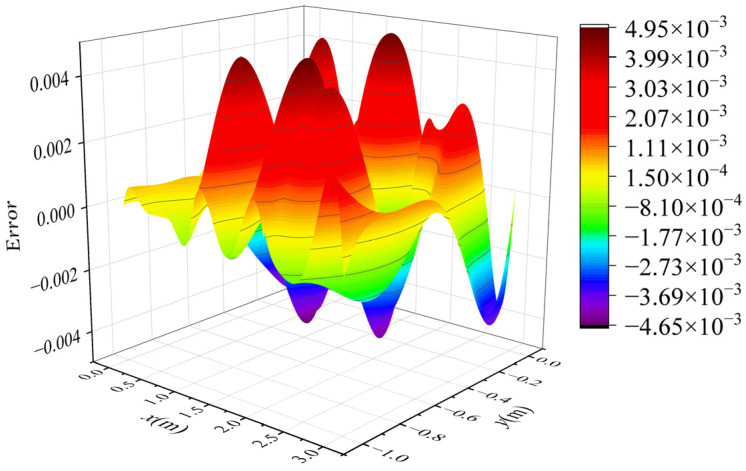
Error distribution of results based on the improved error indicator.

**Figure 5:**
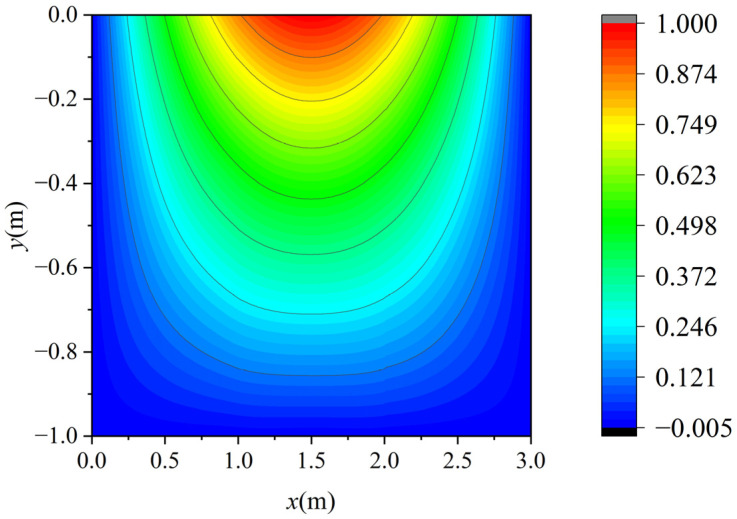
Contour of potential using the proposed method.

The voltage potential of a 2-D square metal box was computed using the proposed method. The square metal box was 3 m long and 1 m wide. The width of the background grid *w* was 1 m, and *l_0_* was 1/3 m. Figure 3 demonstrates the background grids and initial and final node distribution. As shown in Figure 3a, three background grids were used and 40 initial nodes were obtained using the initial node-generation method. In Figure 3b, the red points represent the marked nodes, while the points marked *x* represent the added nodes through iterations. Figure 4 shows the error distribution of results obtained by the proposed method. Figure 5 displays the contour of voltage potential using the automatic adaptive scheme, demonstrating that the proposed method can obtain the voltage potential in the computational domain.

Table 1 provides the key parameters in iterative processes. In Table 1, *i* stands for the number of iterations, *ê_max_* represents the maximum error among all current nodes calculated by Equation (12), and *error_th* denotes the dynamic error threshold for each iteration. As shown in Table 1, the maximum error decreases as the additional nodes are added, indicating that the proposed refinement algorithm can generate proper adding nodes to promote the calculation accuracy. Table 2 presents the comparison of the hybrid method in [21], the RBF method in [20], and the proposed method. The analytic solution of this benchmark problem is given in [21]; *E_max_* is the maximum absolute error between the numerical solution and analytic solution. As shown in Table 2, the proposed method not only achieves the lowest *E_max_* with a relatively small number of nodes but also demonstrates significant advantages in terms of both runtime and memory consumption. This highlights the method’s ability to balance accuracy, efficiency, and computational resources effectively.

### 3.2. Static Iron Piece

**Figure 6:**
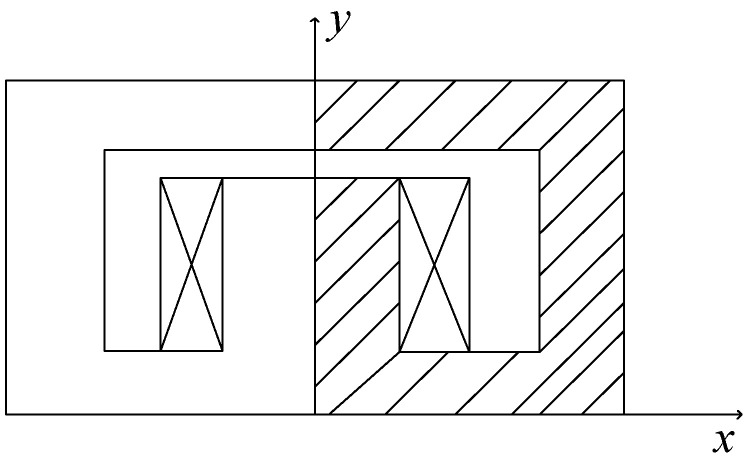
A static iron piece.

The magnetic potential *A_Z_* of a static iron piece was computed using the proposed method. The static iron piece is shown in Figure 6. In the iron piece, the current density in the coil was 250 A/cm^2^ and the permeability of the coil and the iron were *φ*_0_ and 4000 *φ*_0_, respectively [22]. The width of the grid was set as 1 cm, and *l_o_* was 1/2 cm, with a total of 64 background grids. The number of initial nodes was 289 and 7 additional nodes were added to solve this problem.

**Figure 7:**
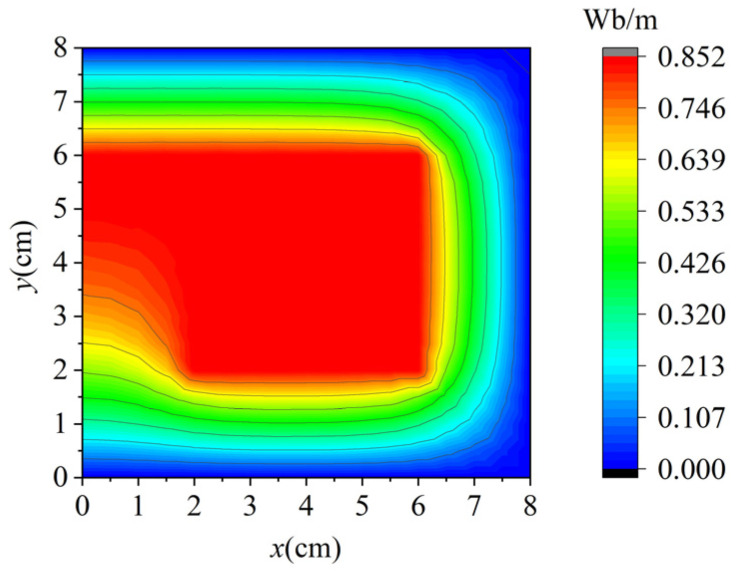
Equivalent lines of vector magnetic potential.

The equivalent lines of vector magnetic potential are given in Figure 7, which demonstrates that the proposed method can obtain the magnetic potential in the computational domain. The maximum error *E_max_*, and condition number and *A_z_* of four nodes are compared in Table 3. *E_max_* was calculated by comparison with the FEM results obtained from commercial software. The coordinates of four nodes were (1,1), (1,2), (1,3), and (0,5), which were selected randomly as represented nodes. From Table 3, it can be seen that the background grids can decrease the condition number and promote the computational accuracy; adding additional nodes can enhance the computational accuracy, but the conditional number also increases. Table 3 demonstrates that the proposed method not only achieves a desired balance between condition number and computational accuracy but also offers significant advantages in terms of both runtime and memory usage. This further underscores the method’s efficiency and effectiveness in handling computational resources while maintaining high accuracy.

**Figure 8:**
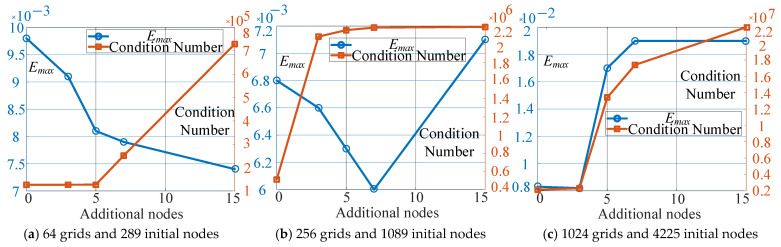
Comparison of calculation results with different parameters.

The number of grids is determined by the width of grid *w*, and the number of nodes is determined by node generation step *l_step_*. A comparison of different numbers of grids, nodes, and additional nodes is given. The parameters were set as follows: *w* = 1 cm, 0.5 cm, and 0.25 cm and *l_step_* = 1/2*w*; therefore, the numbers of girds and nodes were 64 and 289, 256 and 1089, and 1024 and 4225. The number of additional nodes was set as 0, 3, 5, 7, and 15. Figure 8 presents the condition number and the maximum error *E_max_* with different numbers of grids, nodes, and additional nodes. The results show that the condition number was rising as the number of nodes increased. *E_max_* was decreasing with the rising number of nodes when the total number of nodes was relatively small, as shown in Figure 8a,b. However, when the total number of nodes was relatively high, as shown in Figure 8b,c, *E_max_* was increasing with more additional nodes. That is because too many nodes may cause the solving equation to be unstable.

## 4. Conclusions

In this paper, we propose a node generation and adaptive refinement algorithm within the meshless RPIM framework for electromagnetic computations. The simulation results demonstrate that the proposed method effectively generates high-quality nodes, achieving an optimal balance between computational accuracy and complexity. This method has also proved particularly advantageous for simulations in complex electromagnetic environments and multi-scale problems, enabling more accurate analysis of electromagnetic characteristics in sensor design. Its ability to handle intricate geometries and multi-material systems provides significant practical benefits, especially in sensor optimization. Overall, the proposed approach offers a promising tool for enhancing the design and performance of electromagnetic systems in real-world applications.

## Figures and Tables

**Table 1:** Parameters in iterative processes.

*i*	*ê_max_*	No. Nodes	*Error_th*
0	8.677 × 10^−3^	40	6.25 × 10^−3^
1	5.908 × 10^−3^	41	5.67 × 10^−3^
2	5.718 × 10^−3^	42	5.56 × 10^−3^
3	5.410 × 10^−3^	42	----------

**Table 2:** Comparison of different methods.

Algorithm	*E_max_*	Runtime (s)	Memory (MB)	No. Nodes
Hybrid method in [21]	9.80 × 10^−3^	0.84	1.23	40
RBF method in [20]	7.65 × 10^−3^	2.61	2.31	110
The proposed method	4.95 × 10^−3^	0.93	1.87	43

**Table 3:** Comparison of different strategies and FEM.

Method	Nodes	Condition Number	Runtime (s)	Memory (MB)	*E_max_*	*A_z_*1 (Wb/m)	*A_z_*2 (Wb/m)	*A_z_*3 (Wb/m)	*A_z_*4 (Wb/m)
FEM	365	----------	----------	----------	----------	0.3194	0.5899	0.7414	0.8510
Without grids	289	1.5826 × 10^9^	11.23	13.76	1.8 × 10^−2^	0.3163	0.5877	0.7295	0.8331
Without refinemen	289	1.2638 × 10^−5^	2.01	1.97	9.8 × 10^−3^	0.3199	0.5943	0.7378	0.8426
Proposed method	296	2.5120 × 10^−5^	7.39	2.01	7.9 × 10^−3^	0.3207	0.5954	0.7385	0.8431

## Data Availability

No new data were created or analyzed in this study. Data sharing is not applicable to this article.
